# A population-based investigation into inequalities amongst Indigenous mothers and newborns by place of residence in the Northern territory, Australia

**DOI:** 10.1186/1471-2393-12-44

**Published:** 2012-06-09

**Authors:** Malinda Steenkamp, Alice Rumbold, Lesley Barclay, Sue Kildea

**Affiliations:** 1 University Centre for Rural Health North Coast, School of Public Health, University of Sydney, Lismore, Australia; 2 Discipline of Obstetrics and Gynaecology, University of Adelaide, Adelaide and Services Systems and Society Division, Menzies School of Health Research, Darwin, Australia; 3 Australian Catholic University and Mater Medical Research Institute, Women's Health and Newborn Services (Maternity), Mater Health Services, Brisbane, Australia

**Keywords:** Indigenous, Remote, Maternal, Neonatal, Inequalities

## Abstract

****Background**:**

Comparisons of birth outcomes between Australian Indigenous and non-Indigenous populations show marked inequalities. These comparisons obscure Indigenous disparities. There is much variation in terms of culture, language, residence, and access to services amongst Australian Indigenous peoples. We examined outcomes by region and remoteness for Indigenous subgroups and explored data for communities to inform health service delivery and interventions.

****Methods**:**

Our population-based study examined maternal and neonatal outcomes for 7,560 mothers with singleton pregnancies from Australia’s Northern Territory Midwives’ Data Collection (2003–2005) using uni- and multivariate analyses. Groupings were by *Indigenous* status; region (*Top End* (*TE*)*/Central Australia* (*CA*)); *Remote/Urban* residence; and across two large *TE* communities.

****Results**:**

Of the sample, 34.1% were Indigenous women, of whom 65.6% were remote-dwelling versus 6.7% of non-Indigenous women. In comparison to *CA Urban* mothers: *TE Remote* (adjusted odds ratio [aOR] 1.47, 95%CI: 1.13, 1.90) and *TE Urban* mothers (aOR 1.36 (95% CI: 1.02, 1.80) were more likely, but *CA Remote* mothers (aOR 0.43; 95% CI: 0.31, 0.58) less likely to smoke during pregnancy; *CA Remote* mothers giving birth at >32 weeks gestation were less likely to have attended ≥ five antenatal visits (aOR 0.55; 95%CI: 0.36, 0.86); *TE Remote* (aOR 0.71; 95%CI: 0.53, 0.95) and *CA Remote* women (aOR 0.68; 95%CI: 0.49, 0.95) who experienced labour had lower odds of epidural/spinal/narcotic pain relief; and *TE Remote* (aOR 0.47; 95%CI: 0.34, 0.66), *TE Urban* (aOR 0.67; 95%CI: 0.46, 0.96) and *CA Remote* mothers (aOR 0.52; 95%CI: 0.35, 0.76) all had lower odds of having a ‘normal’ birth. The aOR for preterm birth for *TE Remote* newborns was 2.09 (95%CI: 1.20, 3.64) and they weighed 137 g (95%CI: -216 g, -59 g) less than *CA Urban* babies. There were few significant differences for communities, except for smoking prevalence.

****Conclusions**:**

This paper is one of few quantifying inequalities between groups of Australian Indigenous women and newborns at a regional level. Indigenous mothers and newborns do worse on some outcomes if they live remotely, especially if they live in the *TE*. Smoking prevention and high-quality antenatal care is fundamental to addressing many of the adverse outcomes identified in this paper.

## **Background**

Australia’s Aboriginal and Torres Strait Islander (Indigenous) peoples were its original inhabitants. They currently comprise 2.5% of the Australian population [1] and experience substantial disadvantage evident on a range of health and socio-economic indicators [2]. Births to Indigenous mothers account for 3.8% of Australian births. Indigenous maternal and neonatal outcomes have improved in recent decades, but marked inequalities remain between Indigenous and non-Indigenous Australians, with suggestions that for some outcomes (e.g. low birthweight) the disparity is increasing [3-6].

Most Australian literature compares outcomes between Indigenous and non-Indigenous women and infants [7]. This is important to direct policy measures to reduce health inequalities, such as the Australian Government’s *Close the Gap* campaign [8], but it obscures the recognised heterogeneity of Indigenous populations in terms of culture, language, residence, socio-economic circumstances and access to services [2,9,10]. Place of residence (remote community, town, regional city, metropolitan city) is important to the context of Indigenous lives and exerts a major influence on health status [9]. A greater proportion of Indigenous births occur in areas considered remote/very remote (26.2% versus 1.9% amongst non-Indigenous mothers) [4].

There is a wealth of evidence about the diversity of rural and remote communities in Australia, and how they shape the delivery of primary health care [11,12]. Australian literature has shown that rural or remote residence affects all newborns’ risk of adverse neonatal outcomes, such as being small-for-gestational age and stillbirth [13,14]. A recent study demonstrated that Indigenous women in remote areas are significantly less likely to have a healthy baby than Indigenous women in regional and urban areas [7]. There is, however, few other studies about possible differentials in maternal and neonatal outcomes within the Indigenous population, in particular between rural/remote areas, either measured at a regional or community level, despite recognition of the need to consider context and culture in the delivery of maternity services [7,15,16]. It is also unclear whether remoteness itself is a risk factor for poor outcomes or a proxy for other risk factors [7].

The aims of this study were two-fold: (a) to use routinely collected midwives data to examine differences in Indigenous maternal and neonatal outcomes by region and remoteness, and to determine whether remoteness independently predicts poor outcomes; and (b) to examine data from two large remote Indigenous communities to determine whether meaningful differences in maternal and neonatal outcomes can be determined at community-level to inform the planning and delivery of services and interventions.

## **Methods**

### **Study design**

We used a population-based cross-sectional design.

### **Setting**

**Figure 1:**
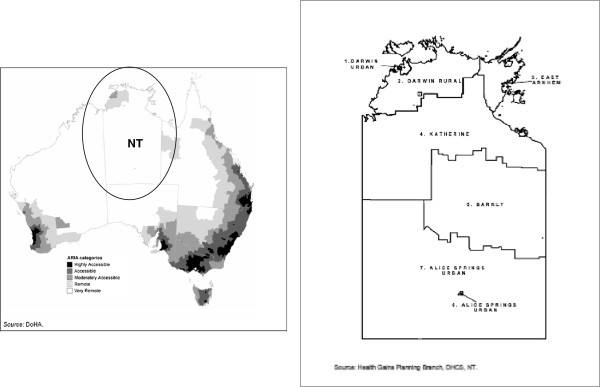
Location of NT in Australia and health districts within the NT as shown in two maps obtained from other publications.

Data examined in this study are from the Northern Territory (NT) of Australia, the third largest federal division area-wise (approximately 1.35 million km^2^) sparsely populated by 1% of the Australian population (230,186 in 2010) [17]. More than 80% of NT Indigenous residents live in areas considered remote or very remote, with the majority living in small communities [18]. Over 100 different traditional Indigenous languages are spoken (the number of speakers ranges from <50 to 3,000). There are seven NT administrative health districts and five regional centres with birthing services (Table 1 and Figure 1) [19-21]. The NT has the highest proportion of Indigenous mothers (36.8% in 2008 compared with <6% in all other jurisdictions) and the highest proportion of births to mothers (Indigenous and non-Indigenous) living in remote or very remote areas (46.2%) [4]. In remote communities, antenatal and infant health services are provided either on-site in community health centres (CHCs); by government-run or community controlled primary health services; and/or by specialist or primary health care outreach services. Current practice is for all remote-dwelling women to be transferred to one of the regional hospitals for birthing at around 38 weeks [22].

**Table 1:** Classification of remoteness for mother’s usual place of residence

**DHF Health District**	**Darwin Urban**^**a**^	**Darwin Rural**	**Katherine**	**East Arnhem**	**Barkly**	**Alice Springs Urban**	**Alice Springs Rural**
**Area**[19]	3,122 km^2^	123,053 km^2^	344,957 km^2^	40,376 km^2^	294,640 km^2^	349 km^2^	545,707 km^2^
**Estimated Resident Population (2004)**[20]							
**Indigenous**	10,682 (9.9%)	10,097 (73.0%)	9,084 (49.2%)	8,813 (63.1%)	3,284 (59.5%)	5,182 (19.0%)	9,744 (77.5%)
**Non-Indigenous**	97,637 (90.1%)	3,728 (27.0%)	9,390 (50.0%)	5,153 (36.9%)	2,239 (40.5%)	22,050 (81.0%)	2,830 (22.5%)
**Total**	108,319 (100%)	13,825 (100%)	18,474 (100%)	13,966 (100%)	5,523 (100%)	27,232 (100%)	12,574 (100%)
**Health service outlets**[19]	4	16	22	13	8	2	32
**Birthing facilities available**[21]	Royal Darwin Hospital (RDH) Darwin Private Hospital (DPH)	Women go to RDH for birth	Katherine District Hospital (KDH)	Gove District Hospital (GDH)	Tennant Creek Hospital (TCH)	Alice Springs Hospital (ASH)	Women go to ASH for birth
**Total number of births per region for 2003-2005**							
**Indigenous**	636 (12.1%)	749 (81.9%)	656 (58.7%)	690 (74.6%)	264 (74.8%)	351 (26.9%)	600 (90.6%)
**Non-Indigenous**	4,614 (87.9%)	165 (18.1%)	461 (41.3%)	235 (25.4%)	89 (25.2%)	955 (73.1%)	62 (9.4%)
**Total**	5,250 (100%)	914 (100%)	1,117 (100%)	925 (100%)	353 (100%)	1,306 (100%)	662 (100%)
**Region**	***TE***	***TE***	***TE***	***TE***	***CA***	***CA***	***CA***
***Remote*/*Urban*status used in this study**[21]	***Urban***	***Remote***	Katherine town – ***Urban*** Remainder of cases - ***Remote***	Nhulunbuy town – ***Urban*** Remainder of cases - ***Remote***	Tennant Creek town - ***Urban*** Remainder of cases -***Remote***	***Urban***	***Remote***
**Comments**	RDH serves as a referral centre for the *TE*, Western Australia and South-East Asia. DPH started operations in 1987. Quality of reporting births to the NTMC stabilised after 1989.				Maternity services were discontinued during 2005 with subsequent births occurring at ASH.	Some ASH cases are referred to Adelaide, South Australia (1,500 km to the south of Alice Springs, Darwin is 1,500 km north of the town.)	

^a^ Includes the capital city, Darwin.

### **Sample**

**Figure 2:**
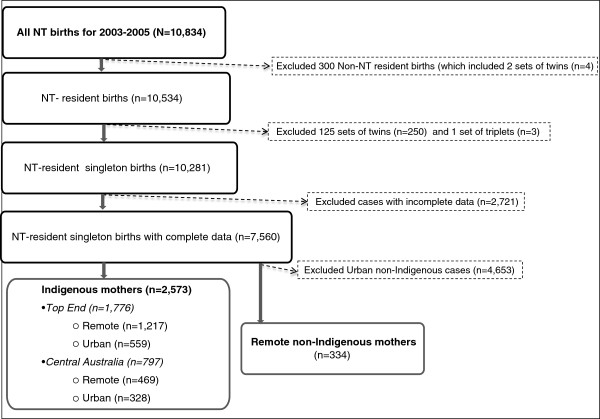
Flow chart of case selection for analysis.

We obtained de-identified data on 10,834 cases from the NT Midwives’ Data Collection (NTMC) for the period 1 January 2003 to 31 December 2005. The NTMC is an electronic dataset of the NT Department of Health (DH) based on data collected by midwives attending the birth. It includes data on all NT mothers and live/stillborn babies with a birthweight ≥400 g and/or a gestation ≥20 weeks. For this analysis, as shown in Figure 2, we excluded 300 births to women whose usual place of residence was interstate/overseas/unknown; 253 twin/triplet births; as well as 2,721 cases with incomplete data for the variables we used in the multivariable analyses (two-thirds of incomplete cases had missing smoking or alcohol data).

### **Comparison groups**

The comparison groups were firstly stratified by Indigenous status. In the NTMC, Indigenous classification is based on self-identification by the mother and consists of four categories: “Aboriginal but not Torres Strait Islander origin”; “Torres Strait Islander but not Aboriginal origin”; “Both Aboriginal and Torres Strait Islander origin”; and “Neither Aboriginal nor Torres Strait Islander origin”. The first three categories are considered as “Indigenous” and the fourth as “non-Indigenous”. In the sample we obtained, cases were either identified as “Neither Aboriginal nor Torres Strait Islander origin” or as “Aboriginal but not Torres Strait Islander origin”. It is likely that the sample included Torres Strait Islander mothers and we use the term “Indigenous” to be more inclusive. We compared outcomes for remote-dwelling Indigenous mothers and newborns to those for non-Indigenous mothers and newborns living in remote areas (Table 2).

**Table 2:** Maternal and newborn characteristics and outcomes, by Indigenous status, remoteness, and region; NT 2003-2005

**Maternal and neonatal characteristics**	**NT-resident women (N = 7,560)**					**Indigenous (N = 2,573)**				
	**Non-Indigenous (n = 4,987)**^**a**^		**Indigenous (n = 2,573)**			**Top End (n = 1,776)**		**Central Australia (n = 797)**		**Chi**^**2**^**or Fisher’s Test for trend**
	***Remote***^***A***^**(n = 334)**	***P-value A vs B***	***Remote***^***B***^**(n = 1,686)**	***Urban***^***C***^**(n = 887)**	***P-value B vs C***	***Remote***^***b***^**(n = 1,217)**	***Urban*(n = 559)**	***Remote*(n = 469)**	***Urban*(n = 328)**	
**Maternal:**										
Mean maternal age in years (±SD)[95% Confidence Interval]	28.4 (±5.7) [27.8, 29.1]	*<0.001*	23 (±5.8) [23.1, 23.7]	24.9 (±5.9) [24.5, 25.3]	*<0.001*	23.7 (±5.9) [23.3, 24.0]	24.8 (±5.8) [24.4, 25.3]	22.7 (±5.8) [22.1, 23.3]	25.0 (±6.1) [24.4, 25.7]	..
Maternal age < 20 years	7.8%	*<0.001*	31.1%	19.8%	*<0.001*	28.6%	19.5%	37.5%	20.4%	*<0.001*
Maternal age ≥ 35 years	16.8%	*<0.001*	5.3%	7.1%	*0.062*	5.7%	7.0%	4.3%	7.3%	*0.193*
First pregnancy	37.4%	*0.078*	32.4%	30.6%	*0.328*	31.2%	30.8%	35.5%	30.2%	*0.262*
Fourth + pregnancy	14.7%	*<0.001*	26.8%	24.5%	*0.198*	29.1%	24.7%	20.9%	24.1%	*0.004*
Less than four antenatal visits	6.3%	*0.001*	12.9%	15.2%	*0.099*	9.6%	17.2%	21.3%	11.9%	*<0.001*
Smoking reported at first visit	24.0%	*<0.001*	47.5%	51.2%	*0.076*	54.7%	53.0%	28.8%	48.2%	*<0.001*
Smoking reported at 36 weeks	21.9%	*<0.001*	44.5%	47.6%	*0.142*	52.4%	49.4%	24.1%	44.5%	*<0.001*
Alcohol use reported at first visit	10.5%	*0.522*	9.4%	8.0%	*<0.001*	8.9%	11.8%	10.9%	20.1%	*<0.001*
Alcohol use reported at 36 weeks	5.7%	*0.708*	6.2%	11.6%	*<0.001*	5.9%	8.8%	7.0%	16.5%	*<0.001*
Diabetes in pregnancy^c^	6.5%	*0.009*	12.7%	12.5%	*0.948*	11.0%	10.9%	17.0%	15.5%	*0.017*
Out-of-hospital birth^d^	<5	*0.019*	4.2%	0.7%	*<0.001*	4.9%	<5	2.4%	<5	*<0.001*
Epidural/spinal pain relief	10.8%	*0.003*	6.2%	10.3%	*<0.001*	5.5%	10.6%	8.1%	9.8%	*0.001*
Narcotics for pain relief	20.4%	*0.703*	21.3%	25.5%	*0.016*	21.6%	25.6%	20.5%	25.3%	*0.109*
Augmented labour	24.3%	*0.137*	28.2%	27.5%	*0.697*	26.5%	27.7%	32.8%	27.1%	*0.071*
Induced labour	23.4%	*0.014*	17.6%	18.6%	*0.536*	15.1%	18.3%	24.1%	19.2%	*<0.001*
Vaginal birth^e^	68.7%	*0.863*	68.3%	73.6%	*0.010*	67.8%	72.8%	71.2%	75.0%	*0.029*
Instrumental vaginal birth	7.8%	*0.272*	6.2%	5.2%	*0.312*	6.2%	5.0%	6.2%	5.5%	*0.775*
Caesarean section (CS)	24.0%	*0.661*	25.1%	21.2%	*0.027*	26.1%	22.2%	22.6%	19.5%	*0.048*
Emergency CS as % of all CS	52.5%	*0.002*	70.5%	67.0%	*0.396*	73.5%	62.9%	61.3%	75.0%	*0.027*
Fetal distress recorded	15.2%	*0.087*	20.1%	20.0%	*0.973*	21.2%	22.5%	17.2%	15.5%	*0.096*
Episiotomy performed (vaginal births)	7.2%	*0.695*	7.5%	5.6%	*0.113*	6.7%	5.7%	<5	<5	*<0.001*
3^rd^/4^th^ degree tear (vaginal births)	<5	*0.351*	1.4%	1.1%	*0.714*	1.5%	0.9%	1.1%	1.5%	*0.522*
Mother LOS ≥ 4 days: vaginal births	33.8%	*0.013*	45.0%	23.0%	*<0.001*	53.2%	24.6%	24.6%	20.3%	*<0.001*
Mother LOS ≥ 4 days: CS births	80.0%	*0.052*	88.9%	83.5%	*0.154*	92.1%	85.5%	79.3%	79.7%	*<0.001*
**Neonatal:**										
Stillbirth (Rate per 1,000 births)	<5	*0.489*	7.7/1,000	<5	*0.447*	5.8/1,000	<5	12.8/1,000	<5	*0.389*
Apgar score < 7 at 5 minutes	2.7%	*0.512*	3.6%	3.2%	*0.543*	3.8%	2.9%	3.2%	3.7%	*0.776*
Newborn needed serious resuscitation^f^	10.8%	*0.771*	11.3%	10.0%	*0.316*	11.8%	8.6%	10.0%	12.5%	*0.144*
Preterm birth (<37 weeks gestation)	3.9%	*<0.001*	12.2%	9.1%	*0.020*	12.7%	9.8%	10.7%	7.9%	*0.054*
Low birthweight (<2,500 g)	4.2%	*<0.001*	10.9%	8.6%	*0.061*	11.8%	8.4%	8.5%	8.8%	*0.054*
Admission to special care (SC)	12.0%	*<0.001*	21.7%	20.0%	*0.300*	21.9%	19.0%	21.3%	21.7%	*0.567*

< 5: Less than 5 counts – data not shown.

^a^ This group includes 4,653 urban-dwelling non-Indigenous mothers for whom relevant data is not shown in Table 2.

^b^ This group includes Communities 1 and 2 and is subdivided in Tables 4 and 5.

^c^ Refers to any mention of diabetes in pregnancy, i.e., mother had either pre-existing diabetes or gestational diabetes recorded. We combined the variables pre-existing diabetes and gestational diabetes to describe ‘diabetes in pregnancy’, because of the small numbers of pre-existing diabetes cases (n = 53).

^d^ Refers to planned home-births, births at community health centres, ‘born-before-arrival’ births, births in hostels, etc.

^e^ Includes 98 vaginal breech cases. Most were preterm births and three were out-of-hospital births.

^f^ Refers to resuscitation with mask and bag, external cardiac massage or intubation.

Data were also grouped by *Remote* or *Urban* residence, defined according to the DH [21,23]. In addition to the two largest regional centres, Darwin (the capital) and Alice Springs, the three other regional centres (Katherine, Tennant Creek and Nhulunbuy) were categorised as ‘urban’ to reflect the availability of birthing services (Table 1) [21]. We compared outcomes for remote-dwelling Indigenous mothers and their newborns with those for their urban Indigenous counterparts (Table 2).

Data for were then grouped by region: *Top End* (*TE*) or *Central Australia* (*CA*). The *TE* and *CA* are geographically distinct NT regions (the monsoonal tropical north and the vast southern desert lands) [24]. Data were grouped by collapsing the current DH health districts (Table 1), which was undertaken to ensure large enough numbers for cells and to protect the privacy of individuals in the case of rare outcomes. We compared outcomes for Indigenous mothers and newborns by region and remoteness (Table 2).

### **Remote Indigenous community data**

This paper also reports data from two field sites from an Australian National Health and Medical Research Council-funded project led by LB and SK [25] (Table 3). The field sites were two large *TE Remote* Indigenous communities, similar in population size (estimated 2,200-3,000), distance from Darwin (>500 km), population profiles (young), accessibility (no road access during the wet season, November-April), no alcohol allowed in the community (i.e., a ‘dry’ town) and place of birth (>90% of births at Royal Darwin Hospital (RDH). Transfer to regional centres mostly occurred by plane. Data for the two communities were extracted from the NTMC sample of 7,560 described above.

**Table 3:** **Comparing Indigenous mothers and newborns from two remote*TE*communities with other*TE Remote*Indigenous mothers and newborns, 2003-2005**

**Maternal and neonatal characteristics**	**Community 1**^**a**^**(n = 105)**	**Community 2**^**a**^**(n = 99)**	**Other*TE Remote***^**a**^**(n = 1,013)**	**Chi**^**2**^**or Fisher’s Test for trend**
**Maternal:**				
Mean maternal age in years (±SD)[95% Confidence Interval]	23.1 (±6.3) [21.9, 24.3]	24.0 (±5.8) [22.8, 25.1]	23.7 (±5.8) [23.2, 24.0]	
Maternal age < 20 years	33.3%	27.3%	28.2%	*0.521*
Maternal age ≥ 35 years	8.6%	5.1%	5.4%	*0.381*
First pregnancy	33.3%	30.3%	31.0%	*0.876*
Fourth + pregnancy	28.6%	25.3%	29.5%	*0.667*
Less than four antenatal visits	6.7%	10.1%	9.9%	*0.600*
Mother reported smoking at first visit	62.9%	42.4%	55.1%	*0.012*
Mother reported smoking at 36 weeks	58.1%	40.4%	53.0%	*0.027*
Mother reported alcohol use at first visit	4.8%	<5	9.8%*	*0.047*
Mother reported alcohol use at 36 weeks	<5	<5	6.8%	*0.05*
Diabetes in pregnancy^b^	8.7%	10.3%	11.3%	*0.879*
Out-of-hospital birth^c^	4.8%	6.1%	4.7%	*0.773*
Epidural/spinal pain relief during labour	4.8%	6.1%	5.5%	*0.936*
Narcotics for pain relief during labour	29.5%	25.3%	20.4%	*0.064*
Augmented labour	29.5%	39.4%	24.9%	*0.006*
Induced labour	13.3%	10.1%	15.8%	*0.278*
Non-instrumental vaginal birth^d^	61.9%	64.7%	68.7%	*0.286*
Instrumental vaginal birth	11.4%	7.1%	5.5%	*0.064*
Caesarean section (CS)	26.7%	28.3%	25.8%	*0.852*
Emergency CS as % of all CS	85.7%	85.7%	70.9%	*0.074*
Fetal distress recorded	19.7%	27.1%	20.8%	*0.439*
Episiotomy performed	20.8%	21.1%	9.4%	*<0.001*
Third/fourth degree tear	<5	<5	1.9%	*0.322*
Mother LOS ≥ 4 days: vaginal births	50.7%	39.4%	55.2%	*0.240*
Mother LOS ≥ 4 days: CS births	100.0%	89.3%	91.6%	*0.227*
**Neonatal:**				
Stillbirth (Rate per 1,000 births)	0	<5	4.9/1,000	*0.152*
Apgar score < 7 at 5 minutes	6.7%	<5	3.7%	*0.216*
Newborn needed serious resuscitation^e^	18.1%	14.1%	11.0%	*0.074*
Preterm birth (<37 weeks gestation)	16.2%	15.2%	12.1%	*0.374*
Low birthweight (<2,500 g)	17.4%	14.1%	11.1%	*0.140*
Admission to special care (SC)	36.2%	28.3%	19.7%	*<0.001*

< 5: Less than 5 counts – data not shown.

^a^ Number shown is percent unless otherwise stated.

^b^ Refers to any mention of diabetes in pregnancy, i.e., mother had either pre-existing diabetes or gestational diabetes recorded.

^c^ Refers to planned home-births, births at community health centres, ‘born-before-arrival’ births, births in hostels, etc.

^d^ Includes vaginal breech cases.

^e^ Refers to resuscitation with mask and bag, external cardiac massage or intubation.

### **Outcomes**

We included two antenatal outcomes: ‘*mothers who gave birth at 32 weeks gestation or more who had five or more antenatal visits*’ and ‘*self-reported smoking in pregnancy*’. Smoking in pregnancy was based on combining ‘*self-reported smoking status at the first antenatal visit*’ and ‘*self-reported smoking status at 36 weeks gestation*’. Smoking in pregnancy was ‘Yes’ if either of these variables were ‘Yes’. There were two intrapartum outcomes: ‘*epidural/spinal and/or narcotic pain relief during labour*’; and a composite maternal measure ‘*normal birth*’ (defined as a pregnancy with a gestational duration of 37 to 41 weeks followed by a vaginal birth with vertex presentation after the spontaneous onset of labour) [26]. Our five neonatal outcomes were: ‘*healthy baby*’ (a composite measure where the newborn was a liveborn singleton of 37–41 completed weeks of gestation with a birth weight of 2,500-4,499 g and an Apgar score at five minutes of seven or more); ‘*preterm birth*’ (<37 weeks gestation at birth) [7]; ‘*low birthweight*’ (LBW, <2.500 g); ‘*special care (SC) admission at birth*’ and ‘*mean birthweight*’ (the only continuous variable). Outcomes were chosen because they were used in other research on perinatal inequalities (healthy baby, preterm birth, LBW, SC admission, birthweight) [7,13]; they form part of six national indicators measuring progress on early child development in the Australian ‘Close the Gap campaign’ which is aimed at reducing inequalities between Indigenous and non-Indigenous Australians (smoking, antenatal visits) [27], they are important issues for remote-dwelling Indigenous mothers (smoking, antenatal visits, preterm birth, LBW, birthweight) [5]; they were found to differ significantly in the univariate analyses (pain relief during labour, type of birth, preterm birth, SC admission – Tables 2 and 3); or any combination of the reasons above.

### **Statistical analysis**

Means and standard deviations were used to summarise continuous data and frequency distributions were used for categorical data. Comparisons of maternal and neonatal variables (Tables 2 and 3) were undertaken using T-, Chi^2^ or Fisher’s exact tests, as appropriate. Logistic regression models were used to predict the probability of the eight binary outcomes of interest. Linear regression was used to predict birthweight. Each model was adjusted for relevant maternal and neonatal risk factors such as age of mother (continuous variable), first pregnancy (Yes/No), >3 pregnancies (Yes/No), smoking in pregnancy (Yes/No), alcohol consumption in pregnancy (Yes/No), diabetes in pregnancy (Yes/No), inadequate number of antenatal visits, i.e., <4 visits (Yes/No), out-of-hospital birth (Yes/No), epidural/spinal pain relief (Yes/No) or augmented labour (Yes/No) (also see footnotes Table 4). These were identified through the analyses in Tables 2 and 3, as well as published literature [7,13,14]. For the regional and remote analyses, *CA Urban* mothers and newborns were the reference groups as they had the best outcomes in general. For the analysis of community data, Indigenous mothers and neonates from the field sites were compared with the remaining *TE Remote* Indigenous mothers and newborns. Data were analysed with Stata/IC 11.1 for Windows (StataCorp LP 2009). We did post hoc power calculations for the analyses in Tables 4 and 5 using ‘*epidural/spinal or narcotic pain relief during labour*’ as this outcome yielded the smallest sample size combinations in both tables. The Power Analysis and Sample Size (PASS) software package version 11.0.8 (NCSS 2011) were used. Logistic regressions for the outcome of choice on a binary independent variable with a sample size of 721 for Table 4 (300 *CA Urban* mothers and 421 *CA remote* mothers) achieved 80% power at a 0.05 significance level to detect a change, but not for the community analysis in Table 5 (913 mothers in the reference group and 91 in Community 2) where the observed effect was relatively small (odds ratio = 1.30 with corresponding power of 21% at 0.05 significance level to detect a change). Further analysis into the logistic regression model for normal birth comparing Community 1 (105 mothers) and the reference group (1,013 mothers), i.e., yielding the largest sample size of 1,118, showed that we achieved a power of 74% to detect the odds ratio as statistically significant (assuming α = 0.05) for this outcome for the comparison groups specified.

**Table 4:** Outcomes for different groups of Indigenous mothers and newborns, NT 2003-2005

**Outcomes**	**Groups**	**Total no.**	**Count and Per cent**^**a**^	**Odds Ratio [95% CI]**^**a**^	**Adjusted odds ratio [95% CI]**^**a**^
**Maternal:**					
Mothers who gave birth at 32+ weeks gestation who had ≥5 antenatal visits^b^	TE *Remote*	1,194	1,030 (86.3%)	1.33 [0.95, 1.84]	1.31 [0.87, 1.98]
	TE *Urban*	551	425 (77.1%)	0.71 [0.50, 1.01]	0.68 [0.44, 1.04]
	CA *Remote*	462	325 (70.4%)	0.50 [0.35, 0.71]	0.55 [0.36, 0.86]
	CA *Urban*	327	270 (82.6%)	1.00	1.00
Mothers who reported smoking in pregnancy^c^	TE *Remote*	1,217	676 (55.6)	1.33 [1.04, 1.58]	1.47 [1.13, 1.90]
	TE *Urban*	559	296 (53.0%)	1.20 [0.91, 1.57]	1.36 [1.02, 1.80]
	CA *Remote*	469	135 (28.8%)	0.43 [0.32, 0.58]	0.43 [0.31, 0.58]
	CA *Urban*	328	159 (48.5%)	1.00	1.00
Mothers who had epidural/spinal or narcotic pain relief during labour^d^	TE *Remote*	1,103	329 (29.8%)	0.68 [0.52, 0.89]	0.71 [0.53, 0.95]
	TE *Urban*	501	202 (40.3%)	1.09 [0.81, 1.46]	1.12 [0.82, 1.53]
	CA *Remote*	421	134 (31.8%)	0.75 [0.55, 1.02]	0.68 [0.49, 0.95]
	CA *Urban*	300	115 (38.3%)	1.00	1.00
Mothers who had a normal birth^e^	TE *Remote*	1,217	605 (49.7%)	0.78 [0.61, 1.00]	0.47 [0.34, 0.66]
	TE *Urban*	559	301 (53.9%)	0.92 [0.70, 1.22]	0.67 [0.46, 0.96]
	CA *Remote*	469	222 (47.3%)	0.71 [0.54, 0.95]	0.52 [0.35, 0.76]
	CA *Urban*	328	183 (55.8%)	1.00	1.00
**Neonatal:**					
Healthy baby^f^	TE *Remote*	1,217	966 (79.4%)	0.78 [0.60, 1.01]	0.79 [0.58,1.10]
	TE *Urban*	559	465 (83.2%)	0.89 [0.62, 1.26]	1.00 [0.64, 1.57]
	CA *Remote*	469	373 (79.5%)	0.79 [0.57, 1.08]	0.83 [0.56, 1.23]
	CA *Urban*	328	267 (81.4%)	1.00	1.00
Newborns born preterm (< 37 weeks gestation)^g^	TE *Remote*	1,217	155 (12.7%)	1.69 [1.10, 2.62]	2.09 [1.20,3.64]
	TE *Urban*	559	55 (9.8%)	1.27 [0.78, 2.07]	1.48 [0.81, 2.70]
	CA *Remote*	469	50 (10.7%)	1.39 [0.84, 2.28]	1.40 [0.75, 2.61]
	CA *Urban*	328	26 (7.9%)	1.00	1.00
Newborns with low birthweight (<2,500 g)^h^	TE *Remote*	1,217	144 (11.8%)	1.38 [0.91, 2.10]	1.07 [0.56, 2.03]
	TE *Urban*	559	47 (8.4%)	0.95 [0.58, 1.54]	0.78 [0.38, 1.60]
	CA *Remote*	469	40 (8.5%)	0.96 [0.58, 1.59]	0.82 [0.38, 1.75]
	CA *Urban*	328	29 (8.8%)	1.00	1.00
Newborns admitted to special care (SC)^i^	TE *Remote*	1,217	266 (21.9%)	1.01 [0.75, 1.36]	1.22 [0.75, 1.97]
	TE *Urban*	559	106 (19.0%)	0.85 [0.61, 1.19]	1.47 [0.87, 2.48]
	CA *Remote*	469	100 (21.3%)	0.98 [0.70, 1.38]	1.11 [0.64, 1.9]
	CA *Urban*	328	71 (21.7%)	1.00	1.00
**Outcome**	**Groups**	**Total no.**	**..**	**Coefficient [95% CI]**	**Adjusted Coefficient[95% CI]**
Newborns mean birthweight (in grams)^j^	TE *Remote*	1,217	..	−179 [−254, −105]	−137 [−216, −59]
	TE *Urban*	559	..	−30 [−113, 54]	15 [−71,101]
	CA *Remote*	469	..	−76 [−162, 11]	−73 [−163, 17]
	CA *Urban*	328	Constant:	3,282 [3,216, 3,349]	3,494 [3,342, 3,646]

^a^ Unless otherwise stated.

^b^ Antenatal visit model limited to mothers who had a pregnancy duration of 32 weeks or more and model adjusted for: age of mother, first pregnancy (Yes/No), four or more pregnancies, and diabetes in pregnancy (Yes/No).

^c^ Smoking in pregnancy refers to any reported smoking during pregnancy and combined ‘smoking at first antenatal visit’ and ‘reported smoking at 36 weeks’. Model adjusted for: age of mother (continuous variable), four or more pregnancies (Yes/No), and reported alcohol use in pregnancy (Yes/No).

^d^ Pain relief model limited to mothers who experienced labour and model adjusted for: age of mother, first pregnancy, four or more pregnancies, and out-of-hospital birth (Yes/No).

^e^ Normal birth model adjusted for: age of mother, first pregnancy, four or more pregnancies, smoking, alcohol, diabetes in pregnancy, inadequate number of antenatal visits (Yes/No), out-of-hospital birth, epidural/spinal pain relief (Yes/No) and augmented labour (Yes/No).

^f^ Healthy baby model adjusted for: age of mother, first pregnancy, four or more pregnancies, smoking, alcohol, diabetes, out-of-hospital birth, and non-instrumental vaginal birth.

^g^ Preterm birth model adjusted for: age of mother, first pregnancy, four or more pregnancies, smoking and alcohol use during pregnancy, diabetes in pregnancy.

^h^Low birthweight (LBW) model adjusted for same variables as preterm birth model, as well as preterm birth (Yes/No).

^i^ SC Admission model adjusted for same variables as LBW model, as well as LBW (Yes/No), Apgar score at 5 minutes of less than 7 (Yes/No), serious resuscitation (Yes/No) and non-instrumental vaginal birth (Yes/No).

^j^ Birthweight (as a continuous variable) model adjusted for age same variables as LBW model.

**Table 5:** **Outcomes for Indigenous mothers and newborns from two*TE*communities and for other*TE*Indigenous mothers and newborns, 2003-2005**

**Outcomes**	**Groups (n = 1,217)**	**Total no.**	**Proportion**^**a**^	**Odds Ratio [95% CI]**^**a**^	**Adjusted odds ratio [95% CI]**^**a**^
**Maternal:**					
Mothers who gave birth at 32+ weeks gestation who had ≥5 antenatal visits^b^	Community 1	104	92 (88.5%)	1.29 [0.69, 2.41]	0.97 [0.49, 1.93]
	Community 2	94	85 (90.4%)	1.58 [0.79, 3.22]	1.98 [0.83, 4.71]
	Reference group	996	853 (85.6%)	1.00	1.00
Mothers who reported smoking in pregnancy^c^	Community 1	105	66 (62.9%)	1.33 [0.88, 2.01]	1.42 [0.94, 2.16]
	Community 2	99	42 (42.4%)	0.58 [0.38, 0.88]	0.62 [0.41, 0.95]
	Reference group	1,013	568 (56.1%)	1.00	1.00
Mothers who had epidural/spinal or narcotic pain relief during labour^d^	Community 1	99	36 (36.4%)	1.42 [0.92, 2.19]	1.47 [0.92, 2.35]
	Community 2	91	31 (34.1%)	1.28 [0.81, 2.03]	1.30 [0.80, 2.13]
	Reference group	913	263 (28.7%)	1.00	1.00
Mothers who had a normal birth^e^	Community 1	105	47 (44.8%)	0.80 [0.54, 1.20]	0.58 [0.34, 0.99]
	Community 2	99	49 (49.5%)	0.97 [0.64, 1.47]	1.00 [0.59, 1.70]
	Reference group	1,013	509 (50.2%)	1.00	1.00
**Neonatal:**					
Healthy baby^f^	Community 1	105	78 (74.3%)	0.74 [0.46, 1.17]	0.63 [0.35, 1.11]
	Community 2	99	81 (81.8%)	1.14 [0.67, 1.96]	1.78 [0.83, 3.88]
	Reference group	1,013	807 (79.7%)	1.00	1.00
Newborns born preterm (< 37 weeks gestation)^g^	Community 1	105	17 (16.2%)	1.40 [0.81, 2.43]	1.63 [0.82, 3.26]
	Community 2	99	15 (15.5%)	1.29 [0.72, 2.31]	0.93 [0.42, 2.08]
	Reference group	1,013	113 (12.1%)	1.00	1.00
Newborns with low birthweight (<2,500 g)^h^	Community 1	105	18 (17.1%)	1.66 [0.97, 2.87]	1.71 [0.69, 4.23]
	Community 2	99	14 (14.1%)	1.33 [0.73, 2.41]	0.84 [0.28, 2.51]
	Reference group^‡^	1,013	112 (11.1%)	1.00	1.00
Newborns admitted to special care (SC)^i^	Community 1	105	38 (36.2%)	2.31 [1.50, 3.53]	1.62 [0.78, 3.37]
	Community 2	99	28 (28.3%)	1.60 [1.01, 2.55]	0.68 [0.28, 1.64]
	Reference group^‡^	1,013	200 (19.7%)	1.00	1.00
**Outcome**	**Groups**	**Total no.**	**..**	**Coefficient [95% CI]**	**Adjusted Coefficient[95% CI]**
Birthweight (in grams)^j^	Community 1	105	..	−134 [−259, −8]	−71 [−197, 54]
	Community 2	99	..	−52 [−181, 77]	−17 [−143, 110]
	Reference group	1,013	..	3,119 [3,080, 3,157]	3,457 [3,270, 3,643]

^a^ Unless otherwise stated.

^b-h, j^ Models adjusted for the same variables as in Table 4.

^i^ SC Admission model adjusted for same variables as in Table 4, as well as the dichotomous variable as to whether the birth occurred in Royal Darwin Hospital or not.

### **Ethical approval**

Ethics approval was granted from the Menzies School of Health Research Human Research Ethics Committee (HREC) and ratified by the University of Sydney’s HREC.

## **Results**

For 2003–2005, we included complete data for 7,560 NT-resident mothers and their singleton newborns. Of these, 2,573 (34.0%) were Indigenous women of whom 1,686 (65.5%) were from remote areas, compared with 6.7% of the 4,987 non-Indigenous mothers. For Indigenous mothers, in the *TE*, 68.5% were from remote areas compared with 58.9% in *CA*.

### **Comparing*Indigenous*and*non-Indigenous*mothers and newborns living in remote areas**

Indigenous mothers living in remote areas more often had worse outcomes for important antenatal risk factors in comparison to remote-dwelling non-Indigenous mothers (Table 2). Notably, the former were more often younger than 20 years (31.1% vs 7.8%, P < 0.001); had four or more pregnancies (26.8% vs 14.7%, P < 0.001); had higher proportions of smoking during pregnancy (e.g., 47.5% vs 24.0%, P < 0.001 at first antenatal visit) and inadequate antenatal visits (12.9% vs 6.3%, P = 0.001). Remote-dwelling Indigenous mothers, in comparison to remote non-Indigenous mothers (Table 2), also had lower proportions of epidural/spinal pain relief during labour (6.2% vs 10.8%, P = 0.003). Babies born to remote-dwelling Indigenous mothers were more often preterm (12.2% vs 3.9%, P < 0.001); of LBW (10.9% vs 4.2%; P < 0.001); and more often admitted to SC (21.7% vs 12.0%) when compared to babies of remote-dwelling non-Indigenous mothers.

### **Comparing*remote*and*urban*Indigenous mothers and newborns**

We found that remote*-*dwelling Indigenous mothers, when compared with urban Indigenous mothers (Table 2), were more often aged <20 years (31.1% vs 19.8%, P < 0.001); had a higher proportion of alcohol use at first antenatal visit (9.4% vs 8.0% P < 0.001) but a lower proportion at 36 weeks (6.2% vs 11.6%, P < 0.001). Compared to urban Indigenous mothers, they had lower proportions of epidural/spinal pain relief during labour (6.2% vs 10.3%, P < 0.001); and non-instrumental vaginal births (68.3% vs 73.6%, P = 0.10). We did not find significant differences in the neonatal outcomes shown in Table 2, except for preterm birth. Babies born to remote-dwelling Indigenous mothers were more often born before 37 weeks compared to those born to urban Indigenous mothers (12.2% vs 9.1%, P = 0.020).

### **Comparisons by region and remoteness for Indigenous mothers and newborns**

Table 2 shows significant differences in most risk factors or outcomes between Indigenous mothers by region and remoteness, except for maternal age ≥35 years, first pregnancy, narcotics for pain relief, augmented labour, instrumental vaginal birth, and 3^rd/^4^th^ degree tears. Proportions for teenage pregnancies were higher for both *TE* and *CA Remote* Indigenous mothers compared with their urban Indigenous counterparts (28.6% and 37.5% versus 19.5% and 20.4%, respectively). Smoking during pregnancy was prevalent in the *TE* (e.g. 54.7% for remote mothers and 53.0% for urban mothers at first antenatal visit) and *CA Urban* areas (48.2% at first visit), but lower for *CA Remote* women (28.8%). *CA Remote* women had the highest proportion of inadequate antenatal visits (21.3%) and *TE Remote* women the lowest (9.6%). About 11% of *TE Urban* mothers had epidural/spinal pain relief during labour, compared to <6% of *TE Remote* mothers, while the proportions for *CA Urban* and *CA Remote* mothers were not that different (9.8% vs 8.1%, respectively). *TE Remote* mothers had the lowest proportion of vaginal births (67.8%) while *CA Urban* mothers had the highest (75.0%). *CA Urban* mothers had the lowest proportion of caesarean section (CS) (19.5%), but of these, 75.0% had emergency CS (i.e., after labour had started). *TE Remote* women had the highest proportion of CS (26.1%) and 73.5% of these were emergency CS. *TE Remote* women had significantly longer lengths of stay for both vaginal and CS births compared to the other three comparison groups in Table 2. There were no significant differences in neonatal outcomes by region and remoteness (Table 2).

Table 4 presents the logistic regression and linear regression models examining the impact of region and remoteness on the four maternal and five neonatal outcomes of interest for Indigenous mothers and newborns using *CA Urban* as the reference groups. Compared to *CA Urban* mothers who gave birth at 32 weeks gestation or more and after adjustment for age of mother, first pregnancy, four or more pregnancies, and diabetes in pregnancy, *CA Remote* mothers (aOR 0.55; 95% CI: 0.36, 0.86) were less likely to have attended five or more antenatal visits during their pregnancy. Both *TE Remote* (aOR 1.47; 95% CI: 1.13, 1.90) and *TE Urban* mothers (aOR 1.36; 95% CI: 1.02, 1.80) were more likely to report smoking tobacco in pregnancy after the model was adjusted for age of mother, multiparity and reported alcohol use, while *CA Remote* women were less likely to smoke during pregnancy (aOR 0.43; 95% CI: 0.31, 0.58) than *CA Urban* women. Both *TE Remote* and *CA Remote* women who experienced labour were less likely to have epidural/spinal or narcotic pain relief than *CA Urban* mothers, aOR 0.71 (95% CI: 0.53, 0.95) and aOR 0.68 (95% CI: 0.49, 0.95), respectively after adjustment for age of mother, first pregnancy, >3 pregnancies, and out-of-hospital births. After adjusting for age of mother, first pregnancy, >3 pregnancies, smoking, alcohol, diabetes, <4 antenatal visits, out-of-hospital birth, epidural/spinal pain relief and augmented labour, *TE Remote* (aOR 0.47; 95% CI: 0.34, 0.66), *TE Urban* (aOR 0.67; 95% CI: 0.46, 0.96) and *CA Remote* (aOR 0.52; 95% CI: 0.35, 0.76) mothers had lower odds of having a ‘normal birth’, compared with *CA Urban* women. *TE Remote* newborns were twice as likely as *CA Urban* newborns to be preterm (aOR 2.09; 95% CI: 1.20, 3.64) after taking into consideration the age of mother, first pregnancy, >3 pregnancies, smoking, alcohol, and diabetes in pregnancy. *TE Remote* babies were also more likely to weigh about 137 g less (95% CI: -216 g, -59 g) than *CA Urban* newborns when the model was adjusted for preterm birth, as well as for the variables included in the preterm birth regression model. There were no significant differences by region and remoteness for healthy baby, LBW and SC admission.

### **Comparing two remote*TE*communities with other*TE remote*women**

There were few significant differences between the outcomes for Indigenous mothers from our two remote Indigenous communities of interest and those for other *TE Remote* women, with the exception of smoking; alcohol use; augmented labour and whether an episiotomy was performed (Table 3). Mothers from Community 2 had lower proportions of smoking (e.g., 42.4% compared with 62.9% for Community 1 and 56.1% for the reference group; P = 0.012). Both communities had low proportions of alcohol use (<5%) while the proportion for the reference group was 10.1% which reflects the fact that both were ‘dry’ towns at the time of data collection. Mothers from Community 2 had a high proportion of augmented labour (39.4%) compared to less than 30% for Community 1 and less than 25% for the reference group (P = 0.006). For the two communties, episiotomies were performed in about 21% of cases compared to <10% for the reference group (P < 0.001). There were no statistically differences for any of the neonatal comparisons.

Table 5 shows the community-level analysis in maternal and neonatal outcomes identified in the logistic regression and linear regression models. There were no statistically significant differences for all of the maternal outcomes. There were no statistically significant differences for the neonatal outcomes, except for admission to SC and mean birthweight. Newborns from both Community 1 (Crude OR 2.31; 95% CI: 1.50, 3.53) and Community 2 (Crude OR 1.60; 95% CI: 1.01, 2.55) appeared to be more likely to be admitted to SC after birth. After adjustment for birth at RDH, age of mother, first pregnancy, >3 pregnancies, smoking, alcohol, diabetes, <4 antenatal visits, birthweight, preterm birth, Apgar score at 5 minutes of <7, serious resuscitation, and normal birth, the aOR for Community 1 was 1.62 (95% CI: 0.78, 3.37) and 0.68 (95% CI: 0.28, 1.64) for Community 2. Newborns from Community 1 weighed on average 134 g (95% CI: -259 g, -8 g) less than the newborns of the reference group. However, this finding did not remain statistically significant after the model was adjusted for age of mother, first pregnancy, >3 pregnancies, smoking, alcohol, <4 antenatal visits and preterm birth.

## **Discussion**

In general, remote-dwelling Indigenous mothers had higher proportions of antenatal risk factors, as well as worse outcomes for some labour characteristics compared to non-Indigenous mothers living in remote areas. Indigenous infants also had worse outcomes than non-Indigenous infants. There were some notable differences between urban- and remote-dwelling Indigenous women. The latter were younger, seemed to receive less pain relief during labour, had higher CS rates and longer hospital stays for vaginal births (mainly due to the air service transporting these remote mothers not allowing newborns <8 days on the plane) than urban Indigenous mothers. There were important differences by region and remoteness for risk factors and outcomes seen amongst Indigenous mothers and newborns. *TE Remote* Indigenous women and newborns appeared to have worse outcomes in general. Our findings seem to suggest that remoteness appears to be an independent risk factor as differences persisted for normal birth, preterm birth and birthweight for *TE Remote* mothers and newborns, even after accounting for risk factors and access to care. Our analyses at community level revealed differences in the prevalence of smoking during pregnancy. In summary, we found that for mothers and newborns in the NT, there is a huge disadvantage in being Indigenous and that for a number of outcomes it is worse to be also living in a remote area, especially a remote area in the *TE*.

Most articles on inequalities focus only on neonatal outcomes, but we included maternal outcomes. Therefore, this paper expands current knowledge on outcomes for Indigenous newborns, as well as on maternal outcomes for Indigenous women. We also quantify the inequalities in health outcomes between groups of Indigenous women and newborns at a regional level and by remoteness and include analyses for communities. The data identify opportunities for health services and clinicians for targeted service delivery and interventions at the regional and local levels. Specific issues are: smoking during pregnancy, teenage motherhood, antenatal attendance and care, pain relief during labour, normal birth, preterm birth and birthweight.

Our findings reinforce the statement that “preventing Aboriginal mothers from smoking during pregnancy is the single most effective short-term intervention to improve Indigenous perinatal outcomes” (p474) [5]. Also, although smoking during pregnancy seemed to be less of a problem in *CA,* the use of chewing tobacco and ‘bush’ tobacco (i.e., wild tobacco plants) [28] is more common in this region [29]. Young Indigenous women in *CA* do not appear to consider these products as harmful during pregnancy [30]. Prevention efforts must incorporate other forms of tobacco use, especially in *CA* in regard to making pregnant women aware of the adverse effects of smokeless tobacco [31,32]. Local context need to be considered in the implementation of smoking interventions as suggested by our analyses for the two communities that showed a difference in prevalence.

In the NT, women become mothers at early ages [33]. Few Australian studies report on whether outcomes for teenage Indigenous mothers differ from those for adult Indigenous mothers; if the majority of Indigenous teenage births occur before or after 17 years of age; and whether those who give birth before 17 years have worse outcomes. Similarly, it is unclear if the outcomes of teenage pregnancy amongst Indigenous women can largely be explained by the prevalence of preventable risk factors such as smoking, remoteness, poorer access to health services, and later presentation for antenatal care. These issues are currently being investigated and are the topic of a subsequent paper.

High-quality antenatal care has been identified as an important strategy to improve Indigenous maternal and neonatal outcomes and closing the gap between Indigenous and non-Indigenous child mortality [34]. In this study, *CA Remote* Indigenous women more often had <4 antenatal visits than *TE Remote* Indigenous women and those in urban areas. However, *TE Remote* Indigenous women and their newborns were more likely to experience adverse outcomes (less ‘normal birth’, more emergency CS, more preterm birth and lower mean birthweight). This suggests that access to antenatal care is not the only important factor, but that the quality of such care matters too [35]. Although strong evidence that the content, frequency and time of antenatal visits are effective is lacking [36], a recent review indicated that programs offering additional antenatal support to mothers at increased risk of having LBW babies may be helpful in reducing the likelihood of antenatal hospital admission and caesarean birth, even though these programs are unlikely to prevent LBW or preterm birth [37]. A study of Indigenous primary health services across Australia identified clear areas for improvement in the delivery of antenatal care [38], For example, only less than 50% of the smokers identified in the study received smoking cessation advice/counselling and just more than half of all women received antenatal education. There were also regional differences in the standard of care, in particular, *CA* services made greater use of antenatal preventative interventions than services in the *TE*[38].

Although improvements in antenatal care are clearly needed, antenatal support by health professionals and others “is unlikely to be powerful enough to overcome the effects of a lifetime of poverty and disadvantage” [37]. Social disadvantage, maternal socio-economic status (SES) and neighbourhood SES have all been linked to adverse birth outcomes [39-41]. Therefore, antenatal care should occur within an integrated primary care model that includes community-based programs, in conjunction with intersectoral interventions in education, housing, and employment [5,16,42].

Remoteness has been described as “a minor but significant factor associated” with poorer Indigenous neonatal outcomes [7]. Our study is consistent with this earlier work, but also demonstrates that remoteness appears to influence maternal outcomes. It is possible that remoteness reflects other unmeasured factors associated with disadvantage. For example, our dataset did not include information on income, education, occupation, and housing circumstances, all of which are likely to be different amongst remote and urban-dwelling individuals, as well as between remote contexts. Also, the distress, social isolation, communication difficulties and practical problems concerning food and transport resulting from taking Indigenous women ‘off country’ to give birth in regional centres [43] are other unmeasured aspects of ‘remoteness’ that are likely to contribute to adverse birth outcomes, such as lower normal birth rates.

A major strength of this study is the data set used: the NT has the highest proportion of Indigenous births of all Australian jurisdictions and there is good identification of Indigenous status [44]. A limitation was that, as the data was de-identified, we could not identify mothers who had multiple pregnancies during the study period. An analysis of a limited set of identifiable NTMC data for 2003–2005 indicated that 13.2% of individual Indigenous mothers had two or more pregnancies during the three-year period. Proportions of mothers who gave birth more than once were similar for the *TE* and *CA*. It was illustrated that this clustering has the potential to lead to incorrect conclusions if results from analyses that assumes independence (e.g., logistic regression) are marginally significant. For example, a p-value of 0.045 or an upper level 95% CI of 0.95 might no longer be significant if clustering was accounted for [45]. High mobility of some mothers could have resulted in misclassification of residence [46]. However, a recent audit of demographic data of NT hospitals showed 88% agreement for patients’ resident health district between hospital records and interview data suggesting that this may not be a major cause of bias in this study [44]. Another limitation is that our definition of ‘remoteness’ differed slightly from the Australian Standard Geographical Classification (ASGC) [47] which do not specifically include birthing services as a consideration. Our study had adequate power to detect statistically significant differences by region and remotes, but small sample sizes impacted the size of effects that were detectable as significant in the community analyses.

Our findings illustrate that context (i.e., remoteness/region/community) should be considered when policy and service delivery decisions are made. This applies to other countries as well. For example, disparities are evident in perinatal care, birth outcomes, and infant health between rural American Indian and Alaska Native persons and rural Whites, despite significant improvements in antenatal care amongst American Indians and Alaska Natives, suggesting that additional measures are needed to close persistent health gaps for this group [48]. Also, a South African study to determine the prevalence and predictors of alcohol exposure during pregnancy found high levels of risk, especially amongst rural women, indicating a need for location-specific prevention programmes [49]. Another study in Northern India reported that community context influenced reproductive wellness [50]. Community-level differences analyses may be useful in identifying important risk factors relevant for service planning and interventions. It is, however, more challenging to show differences in outcomes given the small numbers involved in community-level analysis in the NT.

Future research should concern investigations into whether remoteness is a proxy for particular social and environmental factors in the Australian context. Aspects to consider are summary measures of advantage, disadvantage, economic resources, education and occupation (e.g. the Australian Socio-Economic Indexes for Areas (SEIFA)) [19]; as well as environmental factors in communities (e.g., number of houses per community, state of houses, crowding) and social factors (e.g., community cohesion, level of community violence, level of domestic violence, substance abuse, etc.) [51]. In particular, we suggest that NTMC data be analysed again after the inclusion of SEIFA measures while using ASGC to define remoteness. These investigations were out of scope for the study reported here. There is also an imperative need to explore the distress experienced by remote women giving birth in regional centres and its direct association with adverse birth outcomes.

## **Conclusions**

In conclusion, NT midwives data can be used to meaningfully differentiate subgroups of Indigenous women and infants to identify those with poorer outcomes to target action. It appears that Indigenous mothers and newborns do worse for some outcomes if they live remotely in the *TE*. The prevention of smoking during pregnancy and the delivery of high-quality antenatal care are fundamental to addressing many of the adverse outcomes identified in this paper, especially when it is done in conjunction with community-based programs and broader socio-economic interventions.

## **Abbreviations**

aOR, Adjusted odds ratio; ASGC, Australian Standard Geographical Classification; CA, Central Australia; CHC, Community health centre; CI, Confidence interval; CS, Caesarean section; DH, Department of Health; DPH, Darwin Private Hospital; HREC, Human Research Ethics Committee; LBW, Low birthweight; NT, Northern Territory; NTMC, NT Midwives Collection; RDH, Royal Darwin Hospital; SC, Special care; SEIFA, Socio-economic indexes for areas; TE, Top End.

## **Competing interests**

The authors declare that they have no competing interests.

## **Authors' contributions**

MS conceived the study, organised and conducted the research, obtained ethics approval and the data, executed data and statistical analyses, drafted, critically revised and finalised the manuscript, and is acting as corresponding author. AR participated in the design of the study, the statistical analysis and interpretation of the findings, and helped to draft and critically revise the manuscript. LB and SK participated in the design of the study, helped with the interpretation of the findings and with the draft and revision of the manuscript. All authors read and approved the final manuscript.

## Pre-publication history

The pre-publication history for this paper can be accessed here:

http://www.biomedcentral.com/1471-2393/12/44/prepub
